# Ventricular arrhythmias among patients with implantable cardioverter‐defibrillator during the COVID‐19 pandemic

**DOI:** 10.1002/joa3.12518

**Published:** 2021-02-16

**Authors:** G. Malanchini, P. Ferrari, C. Leidi, G. Ferrari, M. Racheli, M. Senni, P. De Filippo

**Affiliations:** ^1^ ASST Papa Giovanni XXIII Bergamo Italy

## Abstract

**Background:**

Coronavirus Disease‐2019 (COVID‐19) has been associated with myocardial injury and higher risk of arrhythmic complications. However, no reports are available about the effect of the ongoing pandemic on arrhythmias in patients at risk.

**Objective:**

To describe the effect of COVID‐19 pandemic on arrhythmic burden among high‐risk patients.

**Methods:**

This is a cross‐sectional study on the incidence of ventricular arrhythmia (VA) during the pandemic outbreak (study period), compared to the same timeframe in 2019 (reference period). Inclusion criteria were age (>18 years) and having an implantable cardiac defibrillator (ICD).

**Results:**

Among 455 patients enrolled (mean age 64.9 ± 15.7 years; 25.1% females and 39.6% with CRTD), in the study period, 45 (9.9%) patients experienced a total of 86 VA; 8 patients (1.7%) required antitachycardia‐pacing (ATP) and 6 (1.3%) at least one shock. In the reference period, a total of 69 events occurred in 36 patients (7.9%). Six patients (1.3%) required ATP and three (0.7%) at least one shock. The number of patients that suffered from any arrhythmic events in the study period (9.9% vs 7.9%) did not significantly differ from the reference period (χ^2^ = 1.09, *P* = .29). The main predictor of VA during the COVID‐19 pandemic was the previous history of any ICD therapy (OR = 3.84, *P* < .001).

**Conclusions:**

No evidence of an increase of arrhythmic burden was found during the COVID‐19 pandemic among patients with an ICD.

## Graphical abstract

Among 455 patients (mean age 64.9 ± 15.7 years), 25.1% were females and 39.6% had a CRTD. The number of patients that suffered from any arrhythmic events in the study period (9.9 vs 7.9%) did not significantly differ from the reference period (χ^2^ = 1.09, *P* = .29). No evidence of an increase of arrhythmic burden was found during the COVID‐19 epidemic.

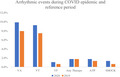

## 1 INTRODUCTION

In December 2019, a new Severe Acute Respiratory Syndrome Coronavirus 2 (SARS‐CoV‐2) emerged as a leading cause of morbidity and mortality in Wuhan, China. Its manifestations known as Coronavirus Disease‐2019 (COVID‐19), suddenly became a major concern for physicians of every specialty.^1^, ^2^

COVID‐19 clinical manifestations are mainly respiratory, but major cardiac complications have been reported in a considerable number of cohorts of hospitalized patients.^3^, ^4^ A more negative outcome was observed among patients admitted to intensive care units in Italy if cardiovascular risk factors (eg, hypertension) were present at baseline.^5^

Studies have linked COVID‐19 to myocardial injury, suggesting that it could lead to a higher risk of arrhythmic complications.^1^

Patients with underlying heart disease, especially those considered at higher risk for cardiac arrhythmia, are routinely implanted, according to Guidelines,^6^, ^7^, ^8^, ^9^ with prophylactic implantable cardioverter‐defibrillator (ICD).

Previous studies have reported seasonal variation, associated with influenza‐virus spread, in the occurrence of ventricular arrhythmias (VA) in patients with an implantable cardiac defibrillator: during high influenza activity periods, patients were more likely to have a VA treated with shock or antitachycardia pacing (ATP).^10^, ^11^

There is a substantial lack of data from a wider population with the history of cardiac diseases on the possible arrhythmogenic effects of the COVID‐19 pandemic. The current study aims to: (a) Characterize the burden of ventricular arrhythmia attributable to COVID‐19 among patients with an ICD during the pandemic outbreak in Italy, focusing on the period between February 21st and April 5th, 2020; and (b) Compare the incidence of VA during the COVID‐19 outbreak to that observed during the same timeframe the previous year (February 21st and April 5th, 2019).

## 2 METHODS

### 2.1 Study design

This was a cross‐sectional comparative study conducted at the Electrophysiology and Cardiac Pacing Unit at ASST Papa Giovanni XXIII Hospital, Bergamo, Italy. The study period was selected based on the epidemiology of the COVID‐19 pandemic in the area,^5^ from February 21st to April 5th, 2020. As a reference period, we selected the same timeframe during the previous year (February 21st – April 5th, 2019).

### 2.2 Sample

**FIGURE 1:**
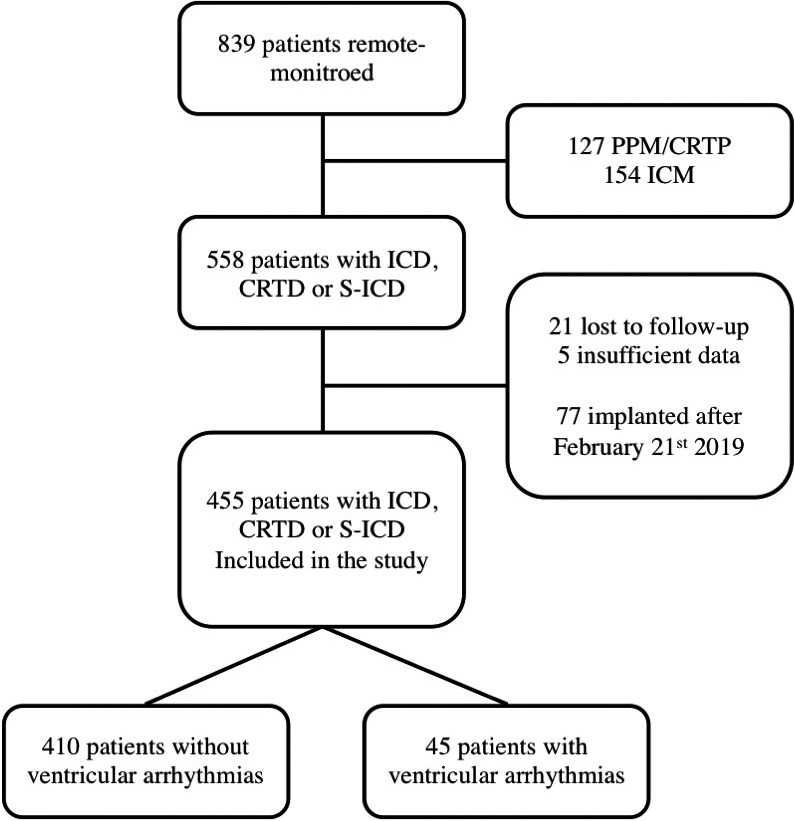
The diagram shows the study population's selection process; ICD, implantable cardioverter‐defibrillator; CRTD, Cardiac resynchronization therapy‐defibrillator; S‐ICD, subcutaneous implantable cardioverter‐defibrillator; PPM, permanent pace‐maker; CRTP, cardiac resynchronization therapy‐ pace‐maker; ICM, implantable cardiac monitor

Inclusion criteria were age (>18 years) and having an implantable cardiac defibrillator (ICD), a subcutaneous ICD (S‐ICD), or cardiac resynchronization therapy defibrillator (CRT‐D). All patients were enrolled in different remote monitoring systems, according to manufacturer technology (BIOTRONIK Home Monitoring, Boston Scientific LATITUDE, and Medtronic CareLink), which allows physicians to access on a secured website to patients’ data. Patients were considered eligible for analysis if available device stored data covered at least 1 month within the study period. Data from the remote‐monitoring system were deidentified before analysis. Patient population was characterized using demographic data including age, gender, device model, reason for implantation, previous history of antitachycardia therapy (shocks or ATP), antitachycardia programming. Among 839 patients with an implanted cardiac device followed up with remote monitoring at the enrolling Institution on April 5th, 2020, 563 had ICD, S‐ICD or CRT‐D. We excluded 26 patients due to: limited follow‐up during the study timeframe (21 patients), unavailable data at remote monitoring (5 patients) or implantation after the study period (77 patients, see Figure 1). The final cohort consisted of 455 patients.

The study was approved by the institutional review board at the enrolling Institution. Every participant gave informed consent for such analysis during hospitalization for device implant or replacement.

### 2.3 Data analysis

The main endpoint of the study was an episode of ventricular tachycardia (VT) or ventricular fibrillation (VF) during the study period (see Supplementary Material).

Programming of detection zones, counters, and discrimination algorithms was set in any patient according to indication, routine practice, and patient's history. Device detected ventricular episodes labeled as VT or VF was adjudicated by two expert cardiac electrophysiologists; in case of discordance a third electrophysiologist was asked to classify the event and the episode was labeled according to the majority vote. Episode data were categorized into binary variables, indicating the presence or absence of a VT, VF, treatment with ATP or with shock during the study period. Data analyses were conducted separately for VA episodes self‐terminating and/or treated with ATP or with at least one shock.

Descriptive statistics for continuous variables were reported as mean (standard deviation) and binary variables as percentages. The event rate was calculated as the number of patients having an event in the study period divided by the total number of patients. Event rate in the study period was compared to that of the reference period using chi‐squared test. The mean number of events in the study period was calculated as the total number of events divided by the total sample size; comparison between periods was carried out by t t test. Univariate and multivariate logistic and linear regressions were conducted in order to examine the associations between variables of interest and the occurrence of an arrhythmic event. In a subgroup of patients, with available data, we also consider physical activity level and heart rate data and compare them to reference period. For all tests, a significance level of 0.05 was used. All analyses were performed using the Stata software version 13.0 (StataCorp, Texas, US).

## 3 RESULTS

### 3.1 Incidence of VA in the study population

As depicted in Figure 1, the final cohort consists of 455 patients; mean age was 64.9 ± 15.7 years. 24.3% of them were females. Table 1 presents patients’ characteristics. More frequent etiologies of heart diseases leading to implant were ischemic heart disease (40.2%), dilated nonischemic cardiomyopathy (32.5%), hypertrophic cardiomyopathy (9.2%), Brugada syndrome (3.5%), and arrhythmogenic right ventricular cardiomyopathy/dysplasia (1.2%).

**TABLE 1:** Patients’ characteristics

	Study population (N = 455)
Age (years)	64.9 ± 15.7
Females	110 (24.3%)
Device type	
ICD – single chamber	163 (35.82%)
ICD – dual chambers	75 (16.48%)
CRTD	180 (39.56%)
S‐ICD	37 (8.13%)
Indication	
Ischemic heart disease	183 (40.22%)
Non‐ischemic dilated cardiomyopathy	148 (32.53%)
Hypertrophic cardiopathy	42 (9.23%)
Arrhythmic disease	55 (12.09%)
Brugada	16 (3.51%)
Syndrome	
Other	39 (8.57%)
Other	27 (5.93%)
Prevention type	
Primary	358 (79.20%)
Secondary	94 (20.80%)
History of previous device therapy	89 (19.56%)
History of recent device therapy (<12 months)	25 (5.49%)
Treatment with antiarrhythmic drugs	
Beta‐blockers	346 (85.86%)
Amiodarone	138 (34.24%)
Mexiletine	14 (3.47%)
Mean lower cut off for tachycardia discrimination (bpm)	182.7 ± 20.9
Mean lower cut off for tachycardia monitor (bpm)	156.2 ± 13.7
Mean cut off for VF discrimination (bpm)	206.7 ± 15.8

Among the study population, 163 patients (35.8%) had a single‐chamber ICD, 75 (16.5%) had a dual‐chamber ICD, 37 (8.1%) a subcutaneous ICD, and 180 patients (39.6%) had a CRTD implanted. Regarding manufacturer the study sample included 80 devices by BIOTRONIK (35 single‐chamber ICD, 3 dual‐chamber ICD, 42 CRTD), 245 by Boston Scientific (103 single‐chamber ICD, 30 dual‐chamber ICD, 75 CRTD, 37 Subcutaneous ICD), and 130 by Medtronic (25 single‐chamber ICD, 42 dual‐chamber ICD, 63 CRTD).

Most patients received a defibrillator for primary prevention (358 patients – 79.2%). Device settings were variably programmed, with 205 (45.4%) with an enabled monitor zone (mean cut‐off value 156.2 ± 13.68 beats per minute – bpm), 18.4% with a VT zone (mean cut‐off value 182.7 ± 20.9 bpm), and 100% having a VF zone (mean cut‐off value 206.6 ± 15.8 bpm).

Within the study cohort, 89 patients (19.6%) had a previous history of device therapy, either ATP or shocks. A vast majority of patients take beta‐blockers as medication (Table 1).

**FIGURE 2:**
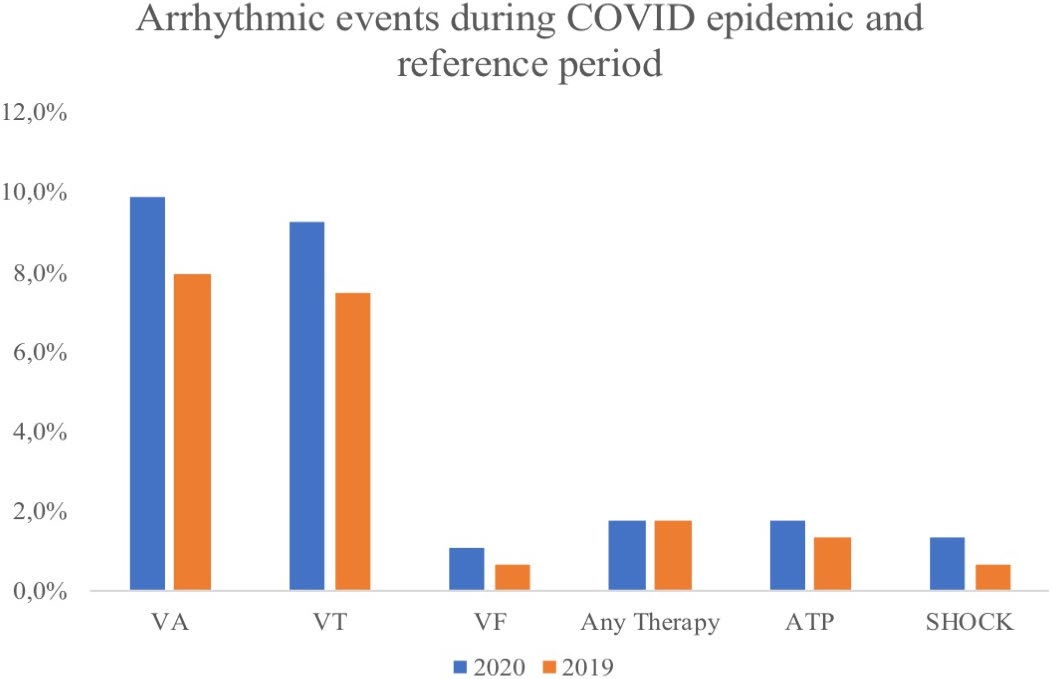
Incidence rates (percentages) of ventricular arrhythmias and ICD therapy among the study population and reference population; VA, ventricular arrhythmias; VT, ventricular tachycardia, VF, ventricular fibrillation; ATP, antitachycardia pacing

Overall, 45 (9.9%) patients experienced from February 21st to April 5th, 2020, a total of 86 VA: 77 episodes (89.5%) of VTs, 9 (10.5%) VF. Eight patients (1.7%) required ATP and six (1.3%) at least one shock during the study period (see Table 1; Figure 2).

During reference timeframe—February 21st to April 5th, 2019—the number of events was 69 VA, experienced by 36 patients (7.9%): 64 episodes (92.7%) of VTs, 5 (7.2%) VF. Six patients (1.3%) required ATP and three (0.7%) at least one shock during the study period. An electrical storm occurred in one patient in the study period, none in the reference period.

### 3.2 Comparison of event rate between study and reference period

The number of patients that suffered from any arrhythmic event did not differ significantly from that of the reference period (χ^2^ = 1.09, *P* = .29). A comparable rate of VT, VF, ATP, and shocks was also found (Table 2).

**TABLE 2:** Comparison of incidence rate in study vs reference period

	Study period (2020) – n = 455	Reference period (2019) – n = 455	χ^2^	*P*
Ventricular Arrhythmias	45 (9.89%)	36 (7.91%)	1.09	.29
Any device Therapy	8 (1.76%)	8 (1.76%)	0.00	1.00
Ventricular tachycardia – VT	42 (9.23%)	34 (7.47%)	0.92	.33
Ventricular fibrillation – VF	5 (1.09%)	3 (0.66%)	0.50	.48
Anti‐tachycardia Pacing – ATP	8 (1.75%)	6 (1.31%)	0.29	.59
ICD shocks	6 (1.31%)	3 (0.66%)	0.41	.52

The overall mean event number per person during the timeframe of the study was 0.19 events/person, not significantly higher than 0.15 events/persons in the reference period (difference 0.04, *P* = .36).

### 3.3 Predictors of VA during the study period

The main predictor of any VA during the COVID‐19 pandemic period was a previous history of any ICD therapy (OR 3.54, *P* < .001) and recent (<12 months) history of ICD interventions either with ATP or shock (OR 9.15, *P* < .001). Age, sex, pharmacologic therapy or secondary prevention indication were not found to be predisposing factors for arrhythmic events during the study period (Table 3 – univariable and multivariable logistic regression analysis).

**TABLE 3:** Logistic regression analysis predicting the risk of ventricular arrhythmia during the COVID‐19 epidemic (February 21st – April 5th, 2020)

Variable	Univariate logistic regressions		Multivariate logistic regression	
	OR	*P*	OR	*P*
Female	1.01	.98	0.87	.75
Age	0.99	.69	1.01	.98
Secondary prevention	0.94	.89		
History of any ICD therapy	3.54	<.001	3.84	<.001
History of recent (<12 months) ICD therapy	9.15	<.001		
Amiodarone	0.81	.55		
Beta‐blockers	0.62	.26		
Mexiletine	1.53	.58	0.69	.60
Etiology				
Ischemic heart disease	0.79	.50		
Non‐ischemic dilated cardiomyopathy	1.53	.19		
Hypertrophic cardiomyopathy	0.24	.17		
Arrhythmic disease	1.50	.35	1.83	.21

Abbreviations: OR, odds ratio.

Among men, the incidence of VA was similar in the study period to the reference period (34 vs 26 events in a sample of 342 males, χ^2^ = 0.034, *P* = .851). The findings were consistent in univariable and multivariable linear regression analysis (Tables S1‐S4).

### 3.4 Activity level and heart rate variations

In a restricted subgroup of patients, devices implanted allow to measure for physical activity level and heart rate: available data show a stable value between the study period and reference period in term of heart rate (68.4 vs 67.7 beats per minute, 1% reduction, net difference 0.7 beats per minute, *P* = 0.53), but a significant reduction of activity level during the study period compared to the reference period (2.46 vs 2.06 h/d, 14.4% reduction, *P* < .001).

## 4 DISCUSSION

The current study found that the incidence rate of any ventricular arrhythmia during the COVID‐19 outbreak acute period was 9.9%. A total of 86 VA was observed, a small number of patients experienced arrhythmia requiring therapy from the ICD: nine patients (1.7%) ATP and eight (1.3%) shock. Importantly, there was no evidence of a significant increase of arrhythmic burden during the COVID‐19 pandemic outbreak, if compared to a reference period, the same timeframe in 2019. The same result was found for sub‐types of VA and for VA requiring ICD therapy. The main predictor of the arrhythmic event during the study period was a previous history of any device therapy, especially in the previous 12 months; this fact reinforces the findings that at the beginning of the COVID‐19 pandemic personal history of arrhythmia remain the strongest risk factor for a subsequent event.

Little is known about COVID‐19 and arrhythmias, especially among patients with an ICD implanted, for whom the risk is generally considered very high. Previous reports of high risk for cardiac damage and arrhythmia refer primarily to very sick populations admitted to hospital for severe acute respiratory syndrome caused by SARS‐CoV2^4^, ^5^, ^12^ and, thus, cannot be generalized to larger spectrum of conditions, during the outbreak of a pandemic. In the series of fatal cases of COVID‐19 from Wuhan, China, a high rate of arrhythmia was reported, leading to hypothesize that COVID‐19 may exacerbate underlying cardiovascular diseases.^12^ However, only a small minority of patients had malignant and fatal cases of such complications.

No data are available on the cardiac effect of COVID‐19 spread among the general population and especially in patients with heart disease. We hypothesized that subjects with an ICD could be the subgroup at higher risk of arrhythmic manifestation of such a pandemic.

It is important to underscore that the Italian area considered in the study, is one with the highest rate of infection and death for COVID‐19,^13^, ^14^ thus a population‐based approach could be considered highly informative.

Since recent reports of higher incidence of cardiac arrest during the COVID‐19 period, also underscore that the rhythm of the presentation was more likely to be asystole or pulseless electrical activity than ventricular rhythms,^15^ our study reinforces the concept that ventricular event is as likely as in previous periods of time.

Considering that hospital reports of cardiac abnormalities among patients with COVID‐19 are limited to subjects admitted to the intensive care unit (ICU), it is unclear how important is the impact of cardiovascular complication among nonhospitalized patients. The presence of myocardial damage, eg, elevated cardiac troponin, in patients with COVID‐19 is reported as independent factors associated with mortality,^16^ but there is a substantial possibility that the overestimation of cardiac involvement in such a condition maybe attributed to studies focusing on very sick patients admitted to ICU with COVID‐19. So that, cardiac involvement maybe less severe among asymptomatic and mildly symptomatic cases, that are missing from most reports.

A growing body of recently published evidence found that seasonal influenza pandemics could trigger acute coronary syndromes and arrhythmias.^10^, ^11^, ^17^ Therefore, it has also been proposed that the increment of underlying cardiovascular diseases may not be specific to COVID‐19, but a more general feature of seasonal viral infections.

The International community is giving high priority to support cardiological practice in a setting of the disruption of previously defined protocols, and, thus, knowledge of epidemiological data is of seminal relevance.^18^, ^19^, ^20^ Previous reports underscore the importance of device derived data analysis in monitoring the activity level of ICD patients,^21^ the present study confirms the sensible reduction of activity level during the pandemic.

The present study showed that, among the unselected consecutive population of patients with an ICD, in a setting of high incidence of disease caused by SARS‐CoV2,^22^ the rate of ventricular arrhythmias was not significantly higher. Possible speculative hypothesis of the stable incidence in the two periods maybe also be related to relatively stable conditions of cardiac patients at the very beginning of the disruptive changing in health‐care providing that occurred in the period, which may become more relevant in the subsequent months and years.^23^ Continuous monitoring of the phenomenon will give in the future more information about the effect of the COVID‐19 pandemic on cardiac health, including arrhythmic burden.

A number of limitations should be considered. First, the study approach does not allow us to verify the actual incidence of clinically relevant COVID‐19 in the cohort. Nevertheless, our approach allowed us to verify the phenomenon in a large sample and to account for the effect of asymptomatic infections in one of the areas most affected by the COVID‐19 pandemic. Furthermore, our approach allowed us to assess the impact of the pandemic from a holistic standpoint, which goes beyond the sole effect of viral activity. Second, we considered only patients with an ICD implanted, generally because considered at higher risk of ventricular arrhythmia due to underlying cardiac disease; this group may be one of the more carefully followed‐up by cardiologists and device specialists, as suggested by the high proportion of antiarrhythmic drugs taken by patients in the study. Fourth, we were unable to assess whether all patients suffering from COVID‐19 were treated with the same pharmacological and nonpharmacological therapy.

## 5 CONCLUSIONS

The current study found that the incidence rate of any ventricular arrhythmia during the first 45 days of the COVID‐19 pandemic outbreak was 9.9% and only a minority of patients required therapy from ICD. There was no evidence of a significant increase in the arrhythmic burden during the study period if compared to the same period in the previous year. The main predictor of arrhythmic events was the previous history of any device therapy, especially within the previous 12 months.

## CONFLICT OF INTEREST

All authors report no conflict of interest.

## Supporting information

Table S1‐S4

Supplementary Material
