# Supplementary material for: Ventricular arrhythmias among patients with implantable cardioverter‐defibrillator during the COVID‐19 pandemic
Source: J Arrhythm. 2021 Feb 16;37(2):407–13. doi: 10.1002/joa3.12518 (PMC8014654; doi:10.1002/joa3.12518)
Supplement: Supplementary file 1 — Table S1‐S4 [file JOA3-37-407-s002.docx]

**Table S1 – supplementary material.** Linear regression analysis predicting risk of ventricular arrhythmic events (ventricular tachycardia) during the COVID-19 epidemic (February 21^st^ – April 5^th^ 2020)

|  | Univariate regressions | | Multivariate regression | |
| --- | --- | --- | --- | --- |
| Variable | Beta coeff. | *p* | Beta coeff. | *p* |
| Female | - 0.02 | 0.78 | -0.06 | 0.44 |
| Age | - 0.01 | 0.26 | 0.01 | 0.38 |
| Secondary prevention | - 0.05 | 0.51 |  |  |
| History of any ICD therapy | 0.32 | < 0.001 | 0.31 | < 0.001 |
| History of recent (< 12 months) ICD therapy | 0.62 | < 0.001 |  |  |
| Amiodarone | 0.09 | 0.23 |  |  |
| Beta-blockers | -0.02 | 0.87 |  |  |
| Mexiletine | 0.55 | 0.005 | 0.36 | 0.06 |
| Aetiology  Ischemic heart disease  Non-ischemic dilated cardiomyopathy  Hypertrophic cardiopathy  Arrhythmic disease | 0.03  0.05  - 0.14  - 0.01 | 0.64  0.44  0.19  0.88 | 0.001 | 0.99 |

**Table S2 – supplementary material.** Linear regression analysis predicting risk of ventricular arrhythmic events (ventricular fibrillation) during the COVID-19 epidemic (February 21^st^ – April 5^th^ 2020)

|  | Univariate regressions | | Multivariate regression | |
| --- | --- | --- | --- | --- |
| Variable | Beta coeff. | *p* | Beta coeff. | *p* |
| Female | - 0.14 | 0.60 | -0.01 | 0.69 |
| Age | 0.01 | 0.70 | 0.01 | 0.34 |
| Secondary prevention | 0.02 | 0.95 |  |  |
| History of any ICD therapy | 0.03 | 0.29 |  |  |
| History of recent (< 12 months) ICD therapy | 0.14 | 0.004 | 0.12 | 0.04 |
| Amiodarone | 0.054 | 0.06 |  |  |
| Beta-blockers | -0.02 | 0.69 |  |  |
| Mexiletine | -0.02 | 0.75 | -0.03 | 0.59 |
| Aetiology  Ischemic heart disease  Non-ischemic dilated cardiomyopathy  Hypertrophic cardiopathy  Arrhythmic disease | -0.02  0.03  - 0.02  0.02 | 0.21  0.18  0.63  0.54 | 0.03 | 0.42 |

**Table S3 – supplementary material.** Linear regression analysis predicting risk of ventricular arrhythmic events (events treated with anti-tachycardia pacing) during the COVID-19 epidemic (February 21^st^ – April 5^th^ 2020)

|  | Univariate regressions | | Multivariate regression | |
| --- | --- | --- | --- | --- |
| Variable | Beta coeff. | *p* | Beta coeff. | *p* |
| Female | - 0.05 | 0.26 | -0.04 | 0.38 |
| Age | 0.01 | 0.76 | 0.02 | 0.12 |
| Secondary prevention | -0.03 | 0.42 |  |  |
| History of any ICD therapy | 0.19 | <0.001 | 0.18 | <0.001 |
| History of recent (< 12 months) ICD therapy | 0.71 | <0.001 |  |  |
| Amiodarone | 0.12 | 0.007 | 0.08 | 0.10 |
| Beta-blockers | -0.02 | 0.77 |  |  |
| Mexiletine | 0.31 | 0.01 | 0.18 | 0.14 |
| Aetiology  Ischemic heart disease  Non-ischemic dilated cardiomyopathy  Hypertrophic cardiopathy  Arrhythmic disease | 0.03  -0.07  - 0.05  -0.01 | 0.42  0.98  0.47  0.87 | 0.07 | 0.91 |

**Table S4 – supplementary material.** Linear regression analysis predicting risk of ventricular arrhythmic events (events treated with shock) during the COVID-19 epidemic (February 21^st^ – April 5^th^ 2020)

|  | Univariate regressions | | Multivariate regression | |
| --- | --- | --- | --- | --- |
| Variable | Beta coeff. | *p* | Beta coeff. | *p* |
| Female | - 0.02 | 0.41 | -0.02 | 0.71 |
| Age | 0.01 | 0.97 | 0.01 | 0.37 |
| Secondary prevention | -0.02 | 0.74 |  |  |
| History of any ICD therapy | 0.87 | 0.02 |  |  |
| History of recent (< 12 months) ICD therapy | 0.34 | <0.001 | 0.18 | 0.002 |
| Amiodarone | 0.09 | 0.02 | 0.05 | 0.08 |
| Beta-blockers | -0.07 | 0.21 |  |  |
| Mexiletine | -0.35 | 0.70 |  |  |
| Aetiology  Ischemic heart disease  Non-ischemic dilated cardiomyopathy  Hypertrophic cardiopathy  Arrhythmic disease | -0.02  0.03  - 0.03  0.01 | 0.27  0.17  0.57  0.67 | 0.03 | 0.45 |
