# Supplementary material for: Ventricular arrhythmias among patients with implantable cardioverter‐defibrillator during the COVID‐19 pandemic
Source: J Arrhythm. 2021 Feb 16;37(2):407–13. doi: 10.1002/joa3.12518 (PMC8014654; doi:10.1002/joa3.12518)
Supplement: Supplementary file 2 — Supplementary Material [file JOA3-37-407-s001.docx]

Supplementary material

Ventricular Arrhythmias adjudication process:

- first stage: objective criteria were applied to exclude non-physiological signals and interferences, ventricular and atrial cycle length comparison; atrio-ventricular coupling; stability of ventricular rhythm tachyarrhythmia;
- second stage: each of two independent electrophysiologists give classification on visual analysis. When discordant, a third expert electrophysiologist was asked to classify the event, blinded to previous decision, and the event was labelled as per majority vote.

Adjudicated VA were classified in one of the groups:

1. VT if detected in any VT zone, according to individualized programming, lasting more than 4 beats;
2. VF if detected in the VF zone according to individualized programming;
3. VA requiring ATP, if any ATP was delivered during the episode of VT or VF;
4. VA requiring shock therapy, if any shock was delivered during the episode of VT or VF.

Episodes were excluded if they were categorized after revision as supraventricular tachycardia or if they were thought to be secondary to device misinterpretation or malfunctioning.
